# Time to full enteral feeding and its predictors among very low birth weight (VLBW) neonates admitted to the neonatal intensive care units (NICU) in comprehensive specialized hospitals in Northwest Ethiopia

**DOI:** 10.1186/s12887-024-04719-w

**Published:** 2024-05-28

**Authors:** Hilena Esubalew, Mengistu Abebe Messelu, Bethelihem Tigabu Tarekegn, Aster Tefera Admasu, Nega Nigussie Abrha, Bewuketu Terefe

**Affiliations:** 1 Felege Hiwot Comprehensive Specialized Hospital, Bahir Dar, Ethiopia; 2 College of Medicine and Health Sciences, Department of Nursing, Debre Markos University, Debre Markos, Ethiopia; 3 College of Medicine and Health Sciences, School of Nursing, Department of Pediatrics and Child Health, University of Gondar, Gondar, Ethiopia; 4 College of Medicine and health Sciences, School of Medicine, Department of Biomedical Sciences, University of Gondar, Gondar, Ethiopia; 5 College of Medicine and Health Sciences, School of Nursing, Department of Emergency and Critical Care Nursing, Univiersity of Gondar, Gondar, Ethiopia; 6 College of Medicine and Health Sciences, School of Nursing, Department of Community Health Nursing, University of Gondar, Gondar, Ethiopia

**Keywords:** Enteral feeding, Neonate, Very low birth weight, Cox-regression

## Abstract

**Background:**

Time to full enteral feeding is the time when neonates start to receive all of their prescribed nutrition as milk feeds. Delayed to achieve full enteral feeding had resulted in short- and long-term physical and neurological sequelae. However, there are limited studies to assess the time to full enteral feeding and its predictors among very low birth-weight neonates in Ethiopia. Therefore, this study aimed to assess the time to full enteral feeding and its predictors among very low birth-weight neonates admitted to comprehensive specialized hospitals in Northwest Ethiopia.

**Methods:**

A multi-center institutional-based retrospective follow-up study was conducted among 409 VLBW neonates from March 1, 2019 to February 30, 2023. A simple random sampling method was used to select study participants. Data were entered into EpiData version 4.2 and then exported into STATA version 16 for analysis. The Kaplan–Meier survival curve together with the log-rank test was fitted to test for the presence of differences among groups. Proportional hazard assumptions were checked using a global test. Variables having a p- value < 0.25 in the bivariable Cox-proportional hazard model were candidates for multivariable analysis. An adjusted Hazard Ratio (AHR) with 95% Confidence Intervals (CI) was computed to report the strength of association, and variables having a P-value < 0.05 at the 95% confidence interval were considered statistically significant predictor variables.

**Result:**

The median time to full enteral feeding was 10 (CI: 10–11) days. Very Low Birth-Weight (VLBW) neonates who received a formula feeding (AHR: 0.71, 95% CI: 0.53, 0.96), gestational age of 32–37 weeks (AHR: 1.66, 95% CI: 1.23, 2.23), without Necrotizing Enterocolitis (NEC) (AHR: 2.16, 95% CI: 1.65, 2.84), and single birth outcome (AHR: 1.42, 95% CI: 1.07, 1.88) were statistically significant variables with time to full enteral feeding.

**Conclusion and recommendations:**

This study found that the median time to full enteral feeding was high. Type of feeding, Necrotizing Enterocolitis (NEC), Gestational Age (GA) at birth, and birth outcome were predictor variables. Special attention and follow-up are needed for those VLBW neonates with NEC, had a GA of less than 32 weeks, and had multiple birth outcomes.

## Introduction

The World Health Organization (WHO) has defined Very Low Birth Weight (VLBW) as less than 1500 g, and preterm birth is before 37 completed weeks of gestation [1]. Very low birth weight is a major cause of morbidity and mortality and accounts for about 60% of neonatal deaths [2].

Full enteral feeding (FEF) is when newborn infants receive all of their prescribed nutrition (120–150 ml/kg/day) as milk feeds (human milk or formula) and do not receive any supplemental parenteral fluids or nutrition from birth [3]. Based on the guideline, the recommended time to achieve FEF is 7 days for infants born 1–1.5 kg and 14 days for infants born < 1 kg [4].

Early introduction and rapid achievement of FEF are the priorities in the nutritional management of VLBW neonates to reduce the need for central venous catheters, the risk of infection, liver problems, persistent gut immaturity, and the length of hospital stay [5, 6]. Furthermore, early enteral feeding is better, safer, and easier than parenteral feeding for later cognitive function and a healthy cardiovascular system [7, 8].

In most resource-limited countries, including Ethiopia, the introduction of enteral feeding for VLBW neonates will be delayed after birth due to the fear of complications from clinical conditions [9]. This delay in FEF may diminish the functional adaptation of the gastrointestinal tract and disrupt the patterns of microbial colonization because gastrointestinal hormone secretion and motility are stimulated by milk feeds [10]. Additionally, it also prolongs hospital stays, doubles hospital expenditures, and increases the risk of hospital-acquired infections, which leads to poor health outcomes [11, 12].

According to studies conducted in developed countries, the morbidity and mortality of VLBW neonates can be substantially reduced by optimizing early enteral feeding, particularly the timing of the introduction of milk feeds [13].

Although many efforts have been made to address the poor practice of early initiation of enteral nutrition among neonates in Ethiopia [14], a considerable number of neonates were kept NPO in the first days after birth [15].

Achieving full enteral feeding in VLBW neonates is an important clinical course index targeted for quality of care. Therefore, identifying predictors of time to full enteral feeding has great clinical significance and will help the healthcare provider practice evidence-based interventions, set priorities, monitor health service programmes, and allocate resources within the health sector. However, to the researchers’ knowledge, there are limited studies in Ethiopia to assess the time to FEF and its predictors among VLBW neonates admitted to the Neonatal Intensive Care Unit (NICU). Therefore, the purpose of this study is to assess the time to FEF and its predictors among VLBW neonates admitted to the NICU in comprehensive specialized hospitals in Northwest Ethiopia.

## Methods

### Study design and period

A multi-center institutional-based retrospective follow-up study was conducted among VLBW admitted to the NICU in Comprehensive Specialized Hospitals in Northwest Amhara from March 1, 2019 to February 30, 2023.

### Study area

The study was conducted in comprehensive specialized hospitals in the Amhara region. According to the Amhara Regional Health Bureau’s (ARHB) annual performance report, the region has eight comprehensive specialized hospitals. Of these, five comprehensive specialized hospitals (i.e., University of Gondar, Tibebe Ghion, Felege Hiwot, Debre Markos, and Debre Tabor) are found in Northwest Amhara, Ethiopia. The average number of admissions to the NICU in each hospital per month is 45 neonates. All the included hospitals have level III NICUs, which provide comprehensive care and service for neonates born before 32 weeks of gestation, weigh less than 1000 g, have medical conditions, or need surgery. The NICUs offer a comparable quality of care and are equipped with infusion pumps, radiant warmers, phototherapy devices, and noninvasive hemodynamic monitoring systems. Different healthcare professionals, such as nurses, general practitioners, residents, and paediatricians are working in the NICUs.

### Population

All VLBW neonates who were admitted to the NICUs in the study hospitals were the source population, whereas VLBW neonates who were admitted to the NICUs in the study hospitals during the study period were the study population.

### Eligibility criteria

All VLBW neonates admitted to the NICUs in Comprehensive Specialized Hospitals in Northwest Amhara during the study period were included. Neonates with congenital malformations and never received full enteral feeding because of their clinical conditions (due to GI surgical interventions), and incomplete data for the outcome variable and time variable were excluded from the study.

### Sample size determination

#### Sample size calculation for the first objective

The sample size was calculated by using a single population proportion formula, which is stated as: *n = (Za/2)*^*2*^*p (1-p)*, where;

d^2^

Zα /2 = 1.96 (Z = score corresponds to 95% confidence level).

*P* = 63.4% (Proportion of neonates achieve a full enteral feeding at 14 days according to the study conducted in Hawassa [14]).

d = margin of error (0.05).

N = total population.

n = required sample size.

1.96^2^ × (0.634) x (1-0.634) = 356.

(0.05)^2^

#### Sample size calculation for the second objective

Using STATA software version 16 and the stpower Cox model, the sample size was estimated for the survival analysis through power and sample size estimates.

$$N = \frac{E}{{P(E)}}\,Where\,E\, = \,\frac{{\left( {\frac{{Z\alpha }}{2}\, + \,Z\beta } \right)\,2}}{{\rho (1\, - \,\rho )\,Ln\,(HR)}}2$$

According to the study conducted in governmental hospitals in Addis Ababa, Ethiopia [15], use of antibiotics, HAI, and starting time of feeding were predictors of time to full enteral feeding with an Adjusted Hazard Ratio (AHR) of 2.92, 6.05, and 2.25, respectively (Table 1).

**Table 1:** Sample size determination to assess predictors of time to FEF among VLBW neonates admitted to the NICU in Comprehensive Specialized Hospitals in Northwest Amhara, 2023

Assumptions	Predictor variables	AHR	P(E)	95% CI	Power	Sample size
Power(Z$\beta$) = 0.842 $\text{Z} a/2=1.96,a=0.05$	Use of antibiotics	2.92	0.16	1.96	0.842	190
	Feeding starting time	6.05	0.23	1.96	0.842	47
	HAI	2.25	0.85	1.96	0.842	63

The largest sample size is obtained from the first objective 356. Thus, by adding 15% for possible incomplete charts and medical records lost, the total sample size was **409.**

### Sampling techniques and procedures

A sampling frame was prepared from a registry of neonates admitted to the NICU. After proportional allocation of the sample size to each study hospital, a simple random sampling technique was used to select charts of the study participants. Stata version 16 was used to generate a random sample of 409 neonatal charts’ from a total of 4146 neonatal charts’.

### Operational definitions

#### Censored

VLBW neonates who didn’t develop the outcome of interest (full enteral feeding) like death, transferred out, left against medical advice, or disappeared during the follow-up time.

#### Event

VLBW neonates who reached full enteral feeding during the follow-up period.

#### Full enteral feeding

The newborn receives all of their prescribed nutrition as milk feeds [3].

#### Follow-up time

Time in days that VLBW neonates have spent since the date of admission to the NICU until 18 days.

#### Birth weight

VLBW < 1500 g, LBW 1500–2499 g, normal birth weight 2500–3999 g (35).

#### Gestational age

Very preterm (28–31 weeks), moderate preterm (32–37 weeks), term (> 37 weeks) (35).

### Data collection tools, techniques, and procedures

The English version of the data extraction checklist was developed from the different literatures [14, 16–18]. The data were extracted from the charts of the neonates and the NICU registration logbook using a data extraction tool for the occurrence of the outcome. The checklist contains maternal-related, neonatal-related, and management-related data. The data were collected by three trained BSc neonatal nurses and one supervisor with an MSc degree. All relevant data were collected retrospectively from neonatal registry books and charts.

### Data quality control

Data collectors were trained for one day before the study to ensure consistency and reduce variations between data collectors. Daily communication was made between the principal investigator and the data collectors throughout the data collection. If any ambiguity or incompleteness is discovered during supervision, it is attempted to be resolved before moving on to the next step. The tool was tested, and the obtained data were reviewed for accuracy, completeness, clarity, and consistency before being entered into data entry forms. Reviewed cards were boldly marked to avoid re-review. Data cleaning was checked for any missing values and data errors.

### Data processing and analysis

The collected data were entered into EpiData version 4.2 and then exported to STATA version 16 for analysis. Numerical descriptive statistics were expressed by using the median with an Interquartile Range (IQR), whereas categorical variables were expressed by frequency with a percentage. The Incidence Density Rate (IDR) of recovery was calculated for the entire follow-up period. The Kaplan-Meier survival curve was computed to estimate the median time to full enteral feeding and the overall probability of recovery, and the log-rank test was fitted to test for the presence of differences among groups. Proportional hazard assumptions were checked both graphically using a log (-log) plot and statistically using a Schoenfeld residual test. The model’s fitness was tested by using the Nelson-Aalen cumulative hazard function against the Cox-Snell residual test. Multicollinearity was checked by using the Variance Inflation Factor (VIF), and the mean result was 1.24, which indicates no major multicollinearity. Both bivariable and multivariable Cox proportional hazards regression analyses were used to identify predictor variables. Variables significant at the p-value < 0.25 level in the bivariable Cox-regression analysis were candidates for multivariable analysis. Adjusted Hazard Ratio (AHR) with 95% Confidence Intervals (CI) was computed to evaluate the strength of the association, and variables having a p-value less than 0.05 were considered statistically significant with the time to full enteral feeding.

## Results

A total of 409 charts of VLBW neonates admitted to the NICU were selected. Of these, 402 were reviewed, and 7 charts were not found during chart retrieval. Finally, 392 (95.8%) charts were included in the analysis, and the remaining 10 charts were excluded due to missing the outcome variable and congenital malformation.

### Maternal-related characteristics of the study participants

The mean age of mothers was 27.71 (SD ± 0.32) years, and the majority (90.56%) of them had ANC follow-up. More than two-thirds (68.9%) were delivered by Spontaneous Vaginal Delivery (SVD); about 153 (39.03%) of mothers had a history of preeclampsia; and only 27 (6.89%) of mothers had a history of DM (Table 2).

**Table 2:** Maternal-related characteristics of the neonates admitted to the NICU in comprehensive specialized hospitals in Northwest Amhara, Ethiopia, 2023

Variables	Categories	Outcome		Total (%)
		Event	Censored	
Age of mother in years	< 21	34	17	51 (13.01)
	21–25	96	9	105 (26.79)
	26–30	116	11	127 (36.40)
	31–35	49	7	56 (14.29)
	> 35	47	6	53 (13.52)
ANC follow-up	Yes	317	38	355(90.56)
	No	25	12	37(9.44)
Number of ANC follow-up	≤ 2	74	13	87 (24.51)
	> 2	243	25	268(75.49)
Mode of delivery	SVD	232	38	270 (68.88)
	C/S	110	12	122 (31.12)
Preeclampsia	Yes	137	16	153(39.03)
	No	205	34	239(60.97)
Maternal DM	Yes	24	3	27(6.89)
	No	318	47	365(93.11)
PROM	Yes	160	28	188(47.96)
	No	182	22	204(52.04)
Chorioamnionitis	Yes	47	8	55(14.03)
	No	295	42	337(85.97)

ANC (Antenatal Care), C/S (Cesarean Section), DM (Diabetes Miletus), PROM (Pre-rupture of Membrane), SVD (Spontaneous Vaginal Delivery)

### Neonatal-related characteristics of the study participants

The median gestational age of neonates during birth was 34 (IQR: 31–35) weeks, and almost all (98.98) VLBW neonates were below the age of 7 days during admission. Among the total VLBW neonates admitted to the NICU, more than three-fourths (78.3%) had a single birth outcome. Almost all (99.23%) of the neonates were diagnosed with sepsis, and about 116 (29.59) with NEC (Table 3).

**Table 3:** Neonatal-related characteristics of the neonates admitted to the NICU in comprehensive specialized hospitals in Northwest Amhara, Ethiopia, 2023

Variables	Categories	Outcome		Total (%)
		Event (*N* = 342)	Censored (*N* = 50)	
Age of neonate at admission	≤ 7 days	339	49	388 (98.98)
	> 7days	3	1	4 (1.02)
Sex	Male	172	33	205 (52.3)
	Female	170	17	187 (47.7)
Gestational age at birth	< 32 weeks	85	34	119 (30.36)
	32–37 weeks	257	16	273 (69.64)
Birth outcome	Singleton	265	42	307(78.32)
	Twins	67	8	75(19.13)
	Triplets	10	0	10(2.55)
Congenital anomalies	Yes	1	3	4 (1.02)
	No	341	47	388 (98.98)
APGAR score 1st minute (*N* = 379)	0–6	34	16	50 (13.19)
	7–10	298	31	329 (86.81)
APGAR score 5th minute (*N* = 379)	0–6	7	14	21 (5.54)
	7–10	325	33	358 (94.46)
NEC	Yes	84	32	116 (29.59)
	No	258	18	276 (70.41)
PNA	Yes	13	10	23 (5.87)
	No	329	40	369 (94.13)
Sepsis	Yes	339	50	389 (99.23)
	No	3	0	3 (0.77)
Type of sepsis (*N* = 389)	EONS	333	49	382 (98.2)
	LONS	6	1	7 (1.8)
RDS	Yes	203	45	248 (63.27)
	No	139	5	144 (36.73)
MAS	Yes	9	1	10 (2.55)
	No	333	49	382 (97.45)
Jaundice	Yes	172	20	192 (48.98)
	No	172	28	200 (51.02)
Meningitis	Yes	11	1	12 (3.06%)
	No	331	49	380 (96.94)
HAI	Yes	31	4	35 (8.93)
	No	311	46	357 (91.07)

APGAR (Appearance, Pulse, Grimace, Reflex), EONS (Early Onset of Neonatal Sepsis), HAI (Hospital Associated Infections), MAS (Meconium Aspiration Sydrome), NEC (Necrotizing Enter colitis), LONS (Late Onset of Neonatal Sepsis), PNA (Perinatal Asphyxia), RDS (Respiratory Distress Syndrome)

### Management-related characteristics of the study participants

Nearly all 384 (97.96%) of the participants started enteral feeding before three days of age, and more than half 222 (56.6%) of the VLBW neonates were fed breast milk. About 325 (82.9%) of the neonates received supplemental oxygen during their admission, and almost all 384 (97.96%) of the neonates were treated with antibiotics (Table 4).

**Table 4:** Management-related characteristics of the neonates admitted to the NICU in comprehensive specialized hospitals in Northwest Amhara, Ethiopia, 2023

Variables	Categories	Outcome		Total (%)
		Event (*N* = 342)	Censored (*N* = 50)	
Starting time of feeding	Early (≤ 3 days)	335	49	384 (97.96)
	Late (> 3 days)	7	1	8 (2.04)
Type of feeding	Breast milk	197	25	222 (56.63)
	Formula	68	11	79 (20.15)
	Mixed	77	14	91 (23.22)
Feeding frequency per day	< 8 times	33	19	52 (13.27)
	≥ 8 times	309	31	340 (86.73)
Oxygen support	Yes	276	49	325(82.91)
	No	66	1	67(17.09)
Device used for oxygen (*N* = 325)	INO2	124	8	132(40.62)
	CPAP	152	41	193(59.38)
Antibiotic use	Yes	335	49	384 (97.96)
	No	7	1	8 (2.04)
Duration of Antibiotic use (*N* = 384)	≤ 7 days	103	29	132 (34.38)
	> 7 days	232	20	252 (65.62)

CPAP (Continuous Positive Airway Pressure), INO2 (Intranasal Oxygen), Time to FEF among VLBW neonates admitted to the NICU

**Fig. 1:**
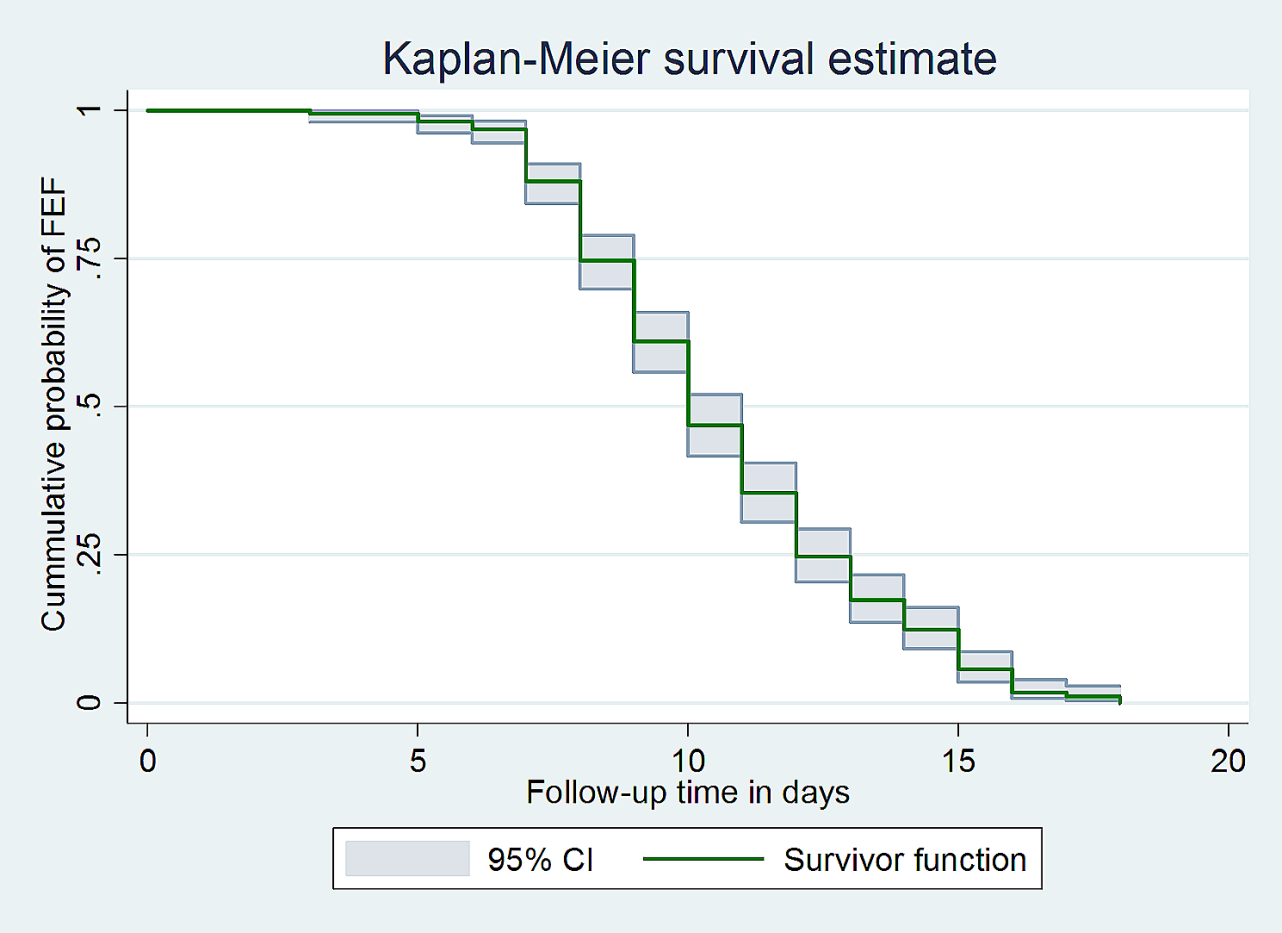
Kaplan-Meier survival estimate for the time to full enteral feeding among VLBW neonates admitted to the NICU in comprehensive specialized hospitals in Northwest Amhara, Ethiopia, 2023

Three hundred ninety-two study participants were followed for a total of 3939 person-days of risk time. The median follow-up time for this study was 10 days (IQR: 8–12), with a minimum of three and a maximum of eighteen days. During the follow-up period, 342 (87.24%) neonates achieved full enteral feeding. Of the total study participants, 1 (0.26%) were on follow-up at the end of the study period, 20 (5.1%) were left against medical advice, and 29 (7.4%) died. The median survival time of full enteral feeding was 10 (CI: 10–11) days (Fig. 1).

### Incidence density rate (IDR) of FEF among VLBW neonates

The overall IDR of full enteral feeding was 8.7 (95% CI: 7.8, 9.7) per 100 person-day observation. The incidence density rate of FEF among neonates at the end of the 7th and 14th days was 1.66 per 100 and 2.16 per 100-person-day observation, respectively.

### Log-rank test result

In addition to the Kaplan-Meier survival estimate, a log-rank test was computed to assess the survival difference between various categorical variables at a p-value of 0.05.

**Fig. 2:**
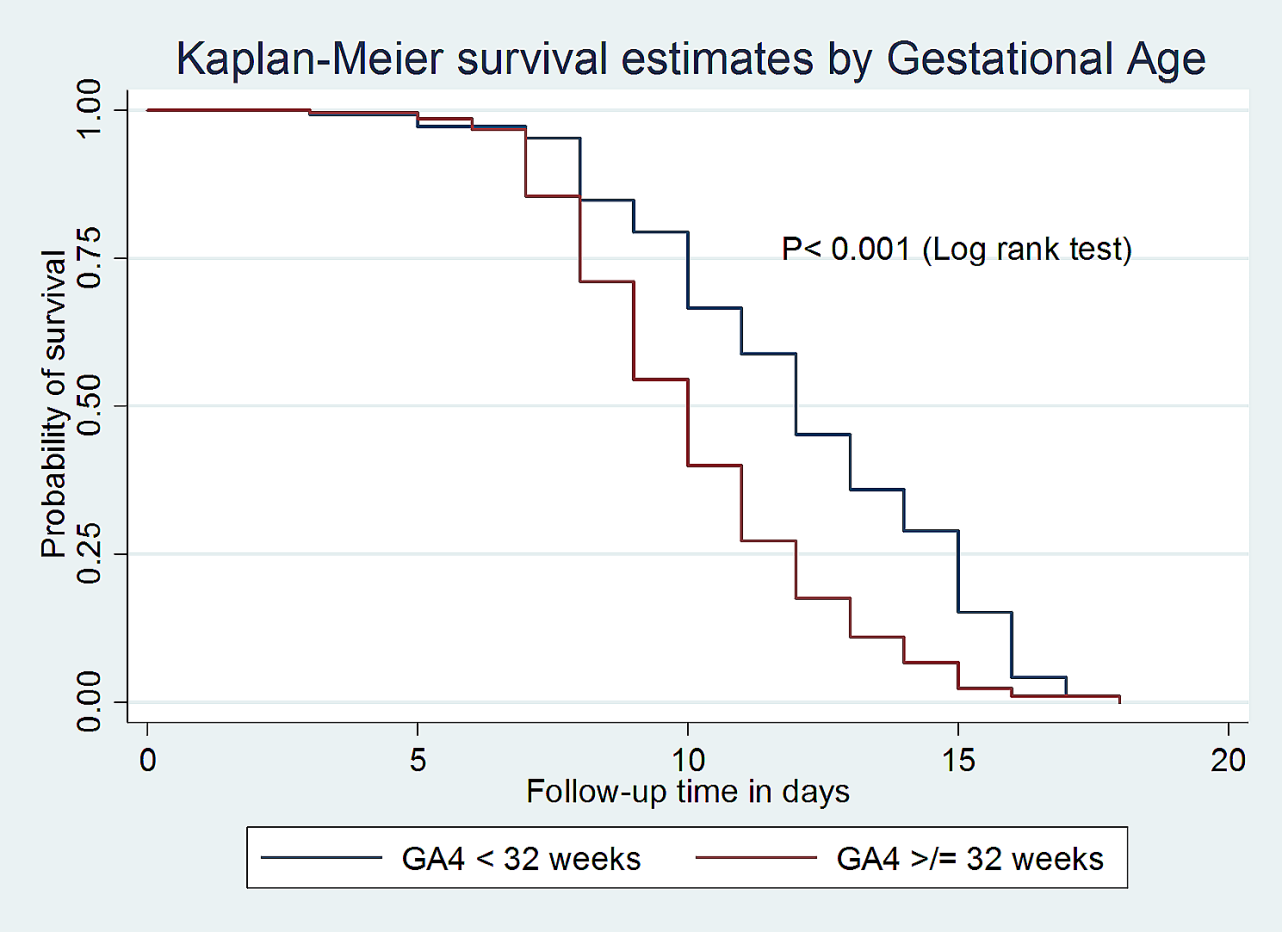
Kaplan-Meier survival estimate by Gestational age for the time to full enteral feeding among VLBW neonates admitted to the NICU in comprehensive specialized hospitals in Northwest Amhara, Ethiopia, 2023

Based on the Kaplan-Meier survival and log rank test, the median survival time to achieve full enteral feeding among VLBW neonates whose GA was 32–37 weeks was 10 days with an IQR of (9–10 days), which is shorter as compared to those who were less than 32 weeks of GA (12 days; IQR: 11–13) with a p-value < 0.001 (Fig. 2).

**Fig. 3:**
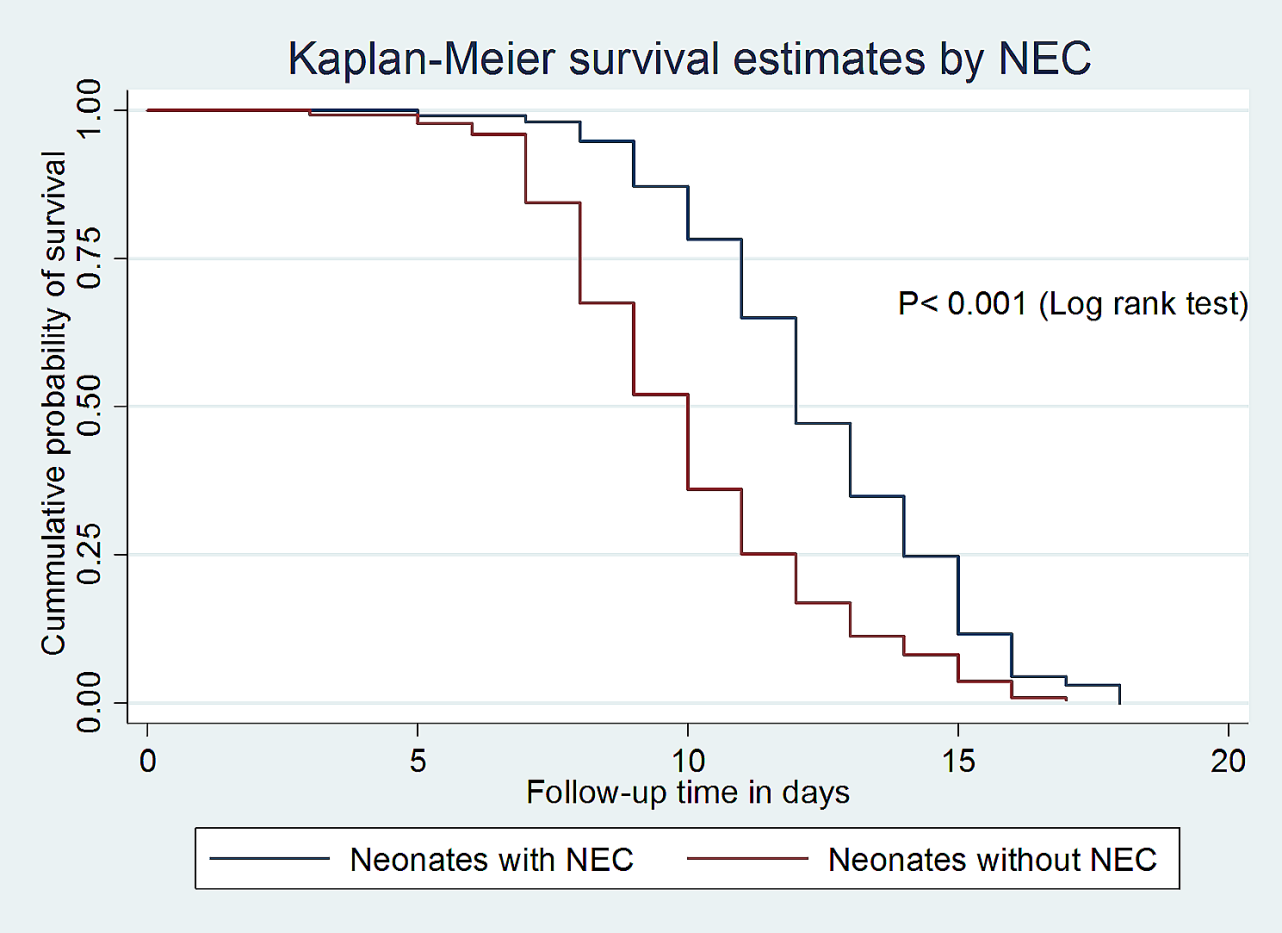
Kaplan-Meier survival estimate by NEC for the time to full enteral feeding among VLBW neonates admitted to the NICU in comprehensive specialized hospitals in Northwest Amhara, Ethiopia, 2023

According to the current study, the median survival time of full enteral feeding among VLBW neonates without NEC was 10 days with an IQR of (9–10 days), which is shorter as compared to those with NEC (12 days; IQR: 12–13) with a p-value < 0.001 (Fig. 3).

**Fig. 4:**
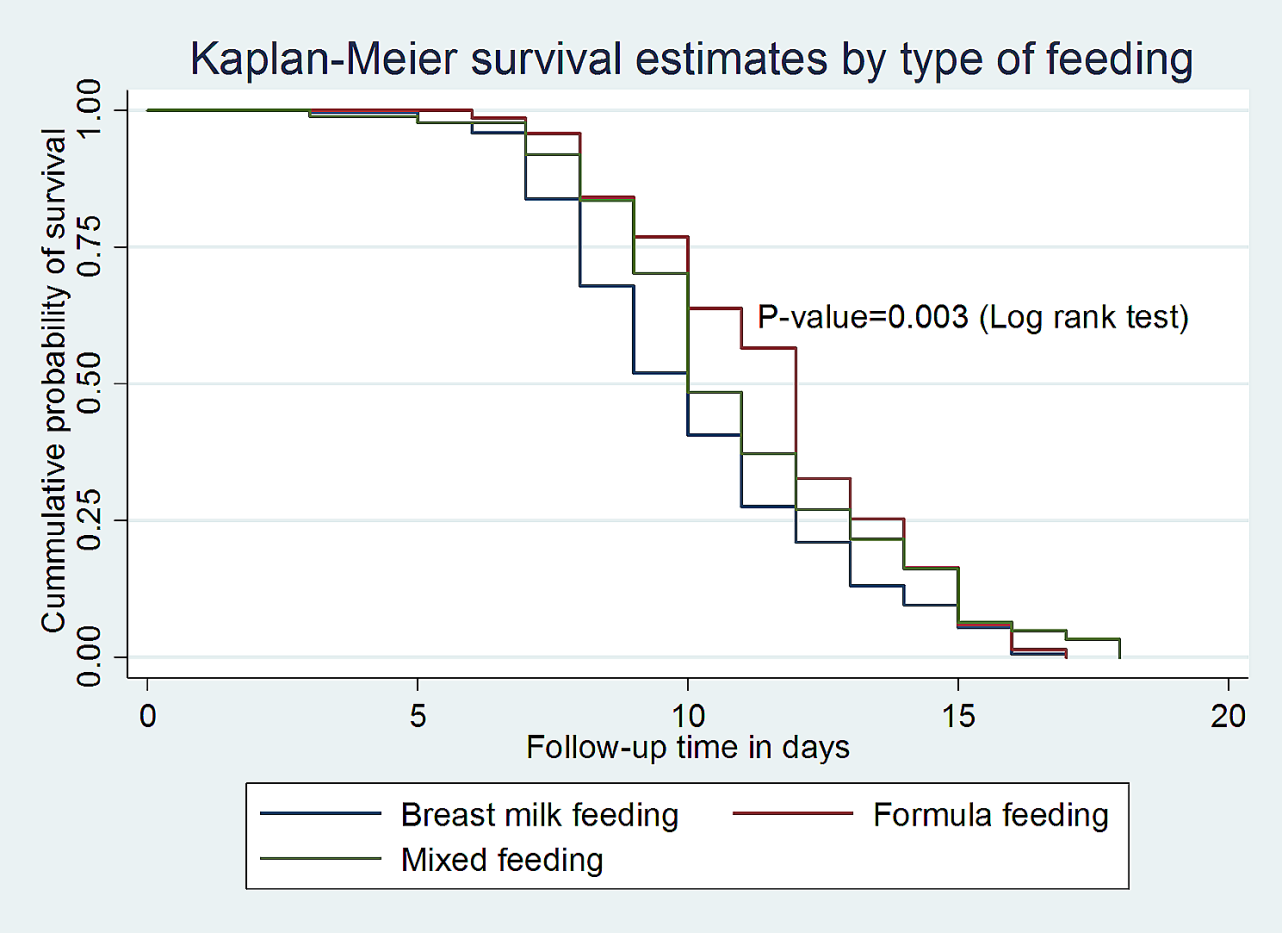
Kaplan-Meier survival estimate by type of feeding for the time to full enteral feeding among VLBW neonates admitted to the NICU in comprehensive specialized hospitals in Northwest Amhara, Ethiopia, 2023

This study found that the median survival time of full enteral feeding among VLBW neonates who fed with formula feeding was 12 days with an IQR of (11–12 days), which is longer as compared to those who fed with breast milk (10 days; IQR: 9–10) with a p-value of 0.003 (Fig. 4).

**Fig. 5:**
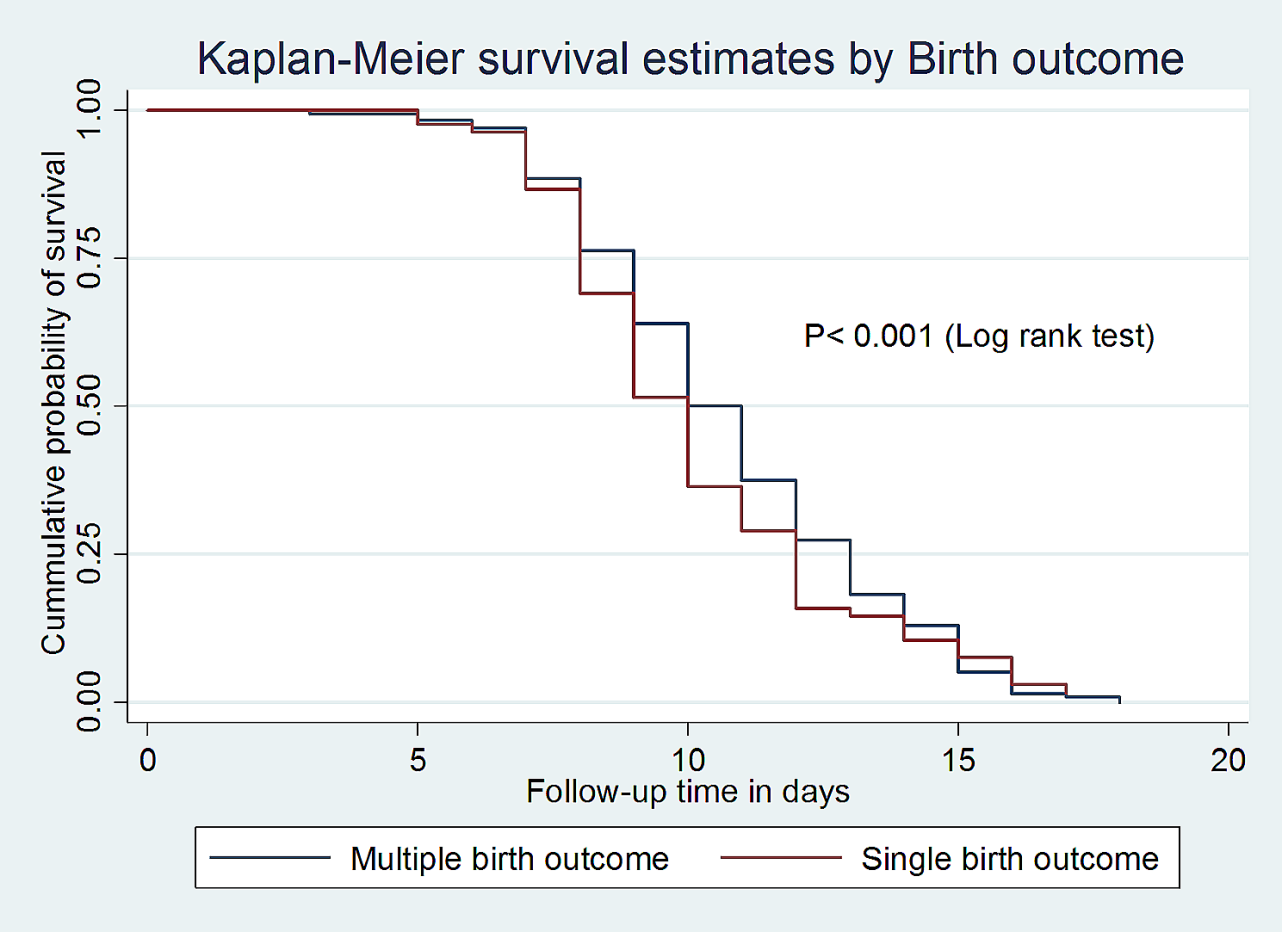
Kaplan-Meier survival estimate by birth outcome for the time to full enteral feeding among VLBW neonates admitted to the NICU in comprehensive specialized hospitals in Northwest Amhara, Ethiopia, 2023

In addition, this study also found that the median survival time of full enteral feeding among VLBW neonates with single birth outcomes was 10 days with an IQR of (9–10 days), which is shorter as compared to those with multiple birth outcomes (11 days; IQR: 10–11) with a p-value < 0.001 (Fig. 5).

### Cox-proportional hazard assumptions

Each variable underwent the Schoenfeld residuals proportional hazard assumption test, and the overall test was done. The p-value was > 0.05 for each variable as well as the overall global test (p-value = 0.4620). This indicates we fail to reject the null hypothesis; it assures that the assumption is satisfied.

### Model goodness of fitness test

**Fig. 6:**
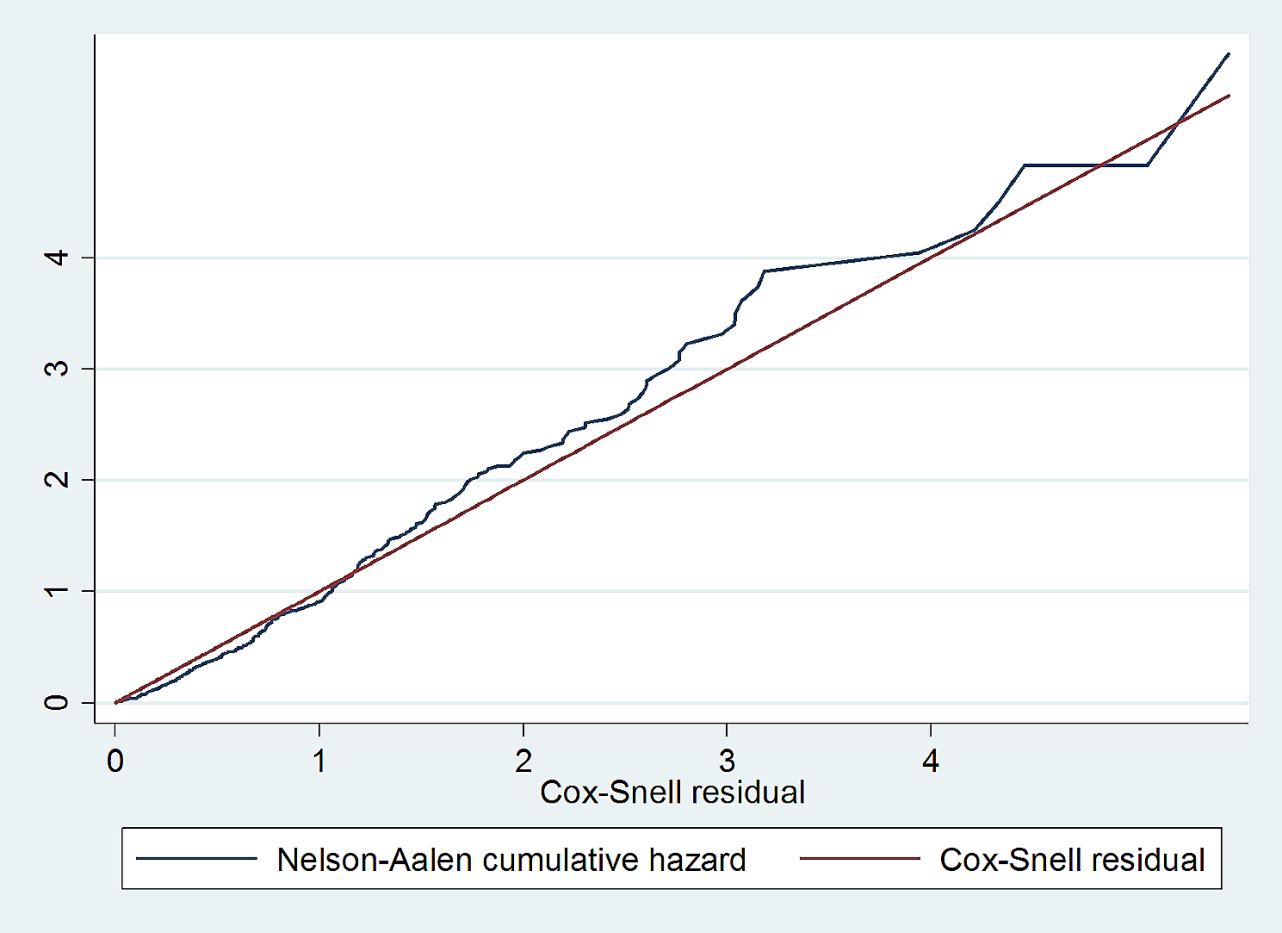
A Cox-Snell residual test to check the model goodness of fit for the assessment of time to full enteral feeding among VLBW neonates, Northwest Amhara, Ethiopia, 2023

The Cox-Snell residual test was employed to test the goodness of fit for the Cox-proportional hazard regression model. The Cox-Snell residuals were estimated based on the Kaplan-Meier estimated survivor function. As shown in the graph below, the cumulative hazard model closely follows a 45-degree against the Cox-Snell residuals line (Fig. 6).

### Predictors of time to full enteral feeding among VLBW neonates

According to the multivariable Cox-proportional hazard regression model, NEC, type of feeding, GA, and birth outcome were statistically significant variables at 5% of the level of significance.

Keeping other variables constant, VLBW neonates who received a formula feeding were less likely to have full enteral feeding by 29% (AHR: 0.71, 95% CI: 0.53, 0.96) as compared with neonates who fed with breast milk. Neonates with a gestational age of 32–37 weeks were 1.7 times (AHR: 1.66, 95% CI: 1.23, 2.23) more likely to have full enteral feeding as compared with those with a gestational age of less than 32 weeks. Furthermore, VLBW neonates without NEC were 2.2 times (AHR: 2.16, 95% CI: 1.65, 2.84) more likely to be fully enterally fed as compared with their counterparts. The hazard of full enteral feeding among VLBW neonates with a single birth outcome was 1.4 times (AHR: 1.42, 95% CI: 1.07, 1.88) higher as compared with their counterparts (Table 5).

**Table 5:** Bivariable and multivariable Cox-regression analysis of predictors of time to full enteral feeding among VLBW neonates admitted to the NICU in Northwest Amhara, Ethiopia, 2023

Variables	Categories	Event	Censored	CHR (95%CI)	AHR (95%CI)	P-value
Gestational age at birth	< 32 weeks	85	34	1	1	
	32–37 weeks	257	16	1.88(1.46, 2.41)	1.66(1.23, 2.23)	**0.001**
NEC	Yes	84	32	1	1	
	No	258	18	2.01(1.56, 2.58)	2.16(1.65, 2.84)	**< 0.001**
Type of feeding	Breast milk	197	25	1	1	
	Formula	68	11	0.69(0.53, 0.92)	0.71(0.53, 0.96)	**0.014**
	Mixed	77	14	0.76(0.59, 0.99)	0.79(0.59, 1.05)	0.110
Birth outcome	Single	265	42	1	1	
	Multiple	77	8	1.17(0.90, 1.50)	1.42(1.07, 1.88)	**0.007**

AHR (Adjusted Hazard Ratio), CHR (Crude Hazard Ration), CI (Confidence Interval), NEC (Necrotizing Enter colitis), PNA (Perinatal Asphyxia), PROM (Premature Rupture of Membrane), RDS (Respiratory Distress Syndrome)

## Discussion

Delay in achieving full enteral feeding is a common problem in VLBW neonates. Delayed time to full enteral feeding directly impacted the postnatal growth and long-term neurodevelopment. Therefore, this study aims to assess the time to full enteral feeding and its predictors among VLBW neonates admitted to the NICU. Therefore, the current study revealed that **t**he median survival time of full enteral feeding was 10 days, and type of feeding, NEC, GA at birth, and birth outcome were statistically significant variables with time to full enteral feeding among VLBW neonates.

This study found that the median time to full enteral feeding was 10 (95% CI: 10–11) days. This finding is higher than the studies conducted in Hawassa (8 days) [14], Addis Ababa (5 days) [15], Nigeria and Kenya (8 days) [17], the UK (8 days), and India (7 days). However, it is lower than the studies done in Italy [19] and the Netherlands [16], which found that the median survival time of full enteral feeding was 13 and 20 days, respectively. The possible reason could be due to the difference in the study population. For instance, the mean gestational age of neonates in the current study was lower than the studies conducted in Italy [19] and the study from the Netherlands conducted among preterm neonates with NEC, which could delay the achievement of full enteral feeding due to its devastating effect on the gastrointestinal tract, whereas the current study includes all VLBW neonates.

Although the NICU guidelines recommend the advancement of enteral nutrition to achieve a full enteral feeding is 7 days for neonates born 1–1.5 kg and 14 days for neonates born < 1 kg [4], the finding of this study is higher. This discrepancy might be due to the difference in the availability of skilled manpower, medical equipment, and laboratory investigations, which directly relate to the quality of services provided in the NICU. Additionally, this might result from a delay in starting enteral feeding, even though WHO recommends that VLBW infants in Low-and Middle-Income Countries (LMICs) should be given 10 ml/kg/day of enteral feeds, preferably starting from the first day of life [20]. Evidence showed that those neonates who delayed achieving FEF had a potential risk for postnatal growth failure, prolonged hospital stays, and poor brain growth [21]. Therefore, many efforts and attempts have been made to achieve full enteral feeding as early as possible.

Keeping other variables constant, VLBW neonates who received a formula feeding were less likely to have full enteral feeding by 29% as compared with neonates who were fed breast milk. This finding was supported by a study conducted in Italy [19]. The reason for this finding might be that human breast milk promotes the maturation of the gut microbiome, which in turn promotes immune modulation, digestion, and the metabolism of nutrients [17]. Additionally, it might be due to the bacterial contamination during formula preparation and feeding, which leads to gastrointestinal disturbances and food intolerance because neonates in the intensive care units are at high risk of developing infections [22]. This implies that exclusive breast feeding for VLBW is essential for the early establishment of full enteral feeding and to prevent complications because breast milk contains numerous bioactive factors, including antioxidants, growth factors, adipokins, and cytokins, as well as unique nutritional factors such as fatty acids and fatty acid derivatives or mediators that serve as an energy source and as regulators of development, immune function, and metabolism [23, 24].

Neonates with a gestational age of 32–37 weeks were 1.7 times more likely to have full enteral feeding as compared with those with a gestational age of less than 32 weeks. The finding is supported by studies done in India [25], Indonesia [26], and Italy [19] which revealed a higher gestational age was associated with an earlier achievement of full enteral feeding. The study conducted in Hawassa, Ethiopia, also supported this finding, which revealed that as GA increases in a week, the time to achieve full enteral feeding decreases by 18.8% [14]. This might be due to differences in physiological maturity among these groups of neonates, in whom feeding intolerance is less common while gestational age increases due to the well-developed and matured gastrointestinal system they have [16]. This in turn reduces the time that the neonates spend on trophic feeding and hospital stays, and reaches full enteral feeding earlier than those who are with small GA. There is also evidence that as the gestational age decreases, there is gastrointestinal and neuromotor immaturity and a deficit in swallowing-sucking coordination, which lead to feeding intolerance problems, causing enteral feeding to be delayed and the time needed to reach full enteral feeding to become longer [17, 22]. Therefore, this evidence would provide an important rationale for quality improvement strategies targeting VLBW neonates with lower gestational ages to initiate full enteral feeding early.

Furthermore, VLBW neonates without NEC were 2.2 times more likely to be fully enterally fed as compared with their counterparts. This finding is supported by the study conducted in neonates in Kenya and Nigeria, which reported that neonates who had NEC or feeding intolerance achieved full enteral feeding as much as 8 days later than those who did not [17]. This could be due to failure in the early initiation of enteral nutrition due to decreased gut development and maturation. Moreover, a well-developed and matured gastrointestinal tract abides by a minute and progressive volume of milk given as a treatment, which also determines the tolerance level of the feeding. The clinical signs and management of NEC necessitate the interruption or discontinuation of feeding in addition to the use of antibiotic therapy and surgical intervention, which would delay the achievement of full enteral feeding [27].

The hazard of full enteral feeding among VLBW neonates who had a single birth outcome was 1.4 times higher as compared with those who had multiple birth outcomes. This is because multiple gestational births are more likely to be associated with prematurity, an increased risk of feeding intolerance, NEC, and infections. Additionally, twin and triplet gestational births are born at a younger gestational age as compared to singletons, and multiple gestational births remain at high risk for various comorbidities such as pulmonary haemorrhage [28]. This implies that VLBW neonates with multiple gestations need a critical evaluation and adjustment of feeding practices based on their level of tolerance.

### Strengths and limitations of the study

This is a multi-center follow-up study with a larger sample size, which increases its validity and generalizability to the target population. However, our study has some limitations. The study design is retrospective, relying on medical records, which may introduce biases due to incomplete or inaccurately recorded information. For instance, clinically important variables used to predict the time to full enteral feeding, such as maternal educational status, income, previous history of failure to achieve FEF, and family size, were not assessed. Moreover, retrospective studies also face limitations in controlling for confounding variables and may not capture the nuances of clinical decision-making in real-time.

### Conclusions and recommendations

According to the current study, the median survival time of full enteral feeding among VLBW neonates admitted to the NICU was high as compared to the guidelines recommendation, which stated that the time to achieve full enteral feeding among VLBW neonates is 7 days. Necrotizing enter colitis, GA at birth, and birth outcome were significantly associated with time to FEF among VLBW neonates admitted to the NICU. A special follow-up and care are needed for those VLBW neonates who had GA less than 32 weeks and multiple birth outcomes, and it is better to focus on prevention and management of NEC among VLBW neonates to decrease the time to achieve FEF. Furthermore, future research is warranted with prospective designs, incorporate socioeconomic factors, and explore the barriers and long-term consequences of delayed enteral feeding on neonatal outcomes.

## Data Availability

Data will be available upon request from the corresponding author.

## Abbreviations

AHR: Adjusted Hazard Ratio
APGAR: Appearance, Pulse, Grimace, Reflex
CHR: Crude Hazard Ratio
CI: Confidence Interval
CPAP: Continues Positive Airway Pressure
FEF: Full Enteral Feeding
HAI: Hospital Associated Infections
HMD: Hyaline Membrane Disease
IDR: Incidence Density Rate
IQR: Inter Quartile Range
KM: Kaplan Meier
LBW: Low Birth Weight
MAS: Meconium Aspiration Syndrome
NEC: Necrotizing Enter colitis
NICU: Neonatal Intensive Care Unit
PNA: Perinatal Asphyxia
PROM: Premature Rupture of Membrane
RDS: Respiratory Distress Syndrome
VLBW: Very Low Birth Weight
